# Minimally Invasive Dentistry: Parent/Carer Perspectives on Atraumatic Restorative Treatments and Dental General Anaesthesia to the Management of Early Childhood Caries

**DOI:** 10.3389/froh.2021.656530

**Published:** 2021-04-23

**Authors:** Peter Arrow, Helen Forrest, Susan Piggott

**Affiliations:** ^1^WA Dental Health Services, Perth, WA, Australia; ^2^Australian Research Centre for Population Oral Health, University of Adelaide, Adelaide, SA, Australia

**Keywords:** child-centred care, child oral health-related quality of life, dental general anaesthesia, early childhood caries, minimal invasive dentistry

## Abstract

**Introduction:** Parents of children treated under dental general anaesthesia (DGA) have reported feelings of concern and anxiety. This study elicited the views of parents/carers (P/C) of children with early childhood caries (ECC) who participated in a randomised trial (core study) which tested the effectiveness of care under DGA or care using alternative minimally invasive Atraumatic Restorative Treatment and the Hall Technique approaches (ART/HT).

**Methods:** P/C of children treated using the ART/HT (test) approach or care under a DGA (control) were interviewed. Focus group semi-structured interviews with P/C were undertaken in community facilities. The transcripts were read and inductively coded into domains to identify emergent themes. The codes were entered into NVivo software to assist data management and were further refined into broad themes.

**Results:** Seven grouped interviews with 14 participants were conducted and one test participant provided a written response. Four groups with eight test participants; two groups with four control participants; and one combined group with one test and one control participant were interviewed. Five broad themes emerged after thematic analysis: (1) Impacts on the child and the family; (2) Child-/family-centred care; (3) Timeliness of care; (4) Affordable care; (5) Accessible care. Impacts were related to that of the effects of the disease, and of the care for the disease. Child-centred/family-centred care (CCC) was a source of appreciation by P/C of both groups when it was experienced. Frustration at the lack of timely care of their child's treatment needs, coupled with the perceived expensiveness of care and difficulties in physically getting to the location for a specialist consultation was expressed by P/Cs in the study.

**Discussion:** The use of the ART/HT enabled the establishment of a relationship between the clinical team and the child and P/C which was central to the delivery of CCC. P/Cs in the DGA arm of the study expressed dissatisfaction more often with the issues of timely care, cost of care and accessibility of care. P/C of both groups were equally satisfied with the treatment, where treatment had been received in a timely, child-centred manner.

**Conclusion:** The findings suggest that minimally invasive approaches which facilitated CCC are acceptable alternative options to the DGA and should be considered for the management of ECC.

**Australian New Zealand Clinical Trials Registry:** ACTRN12616001124426.

## Introduction

Dental caries management in young children with early childhood caries (ECC) can be challenging, and for some children the primary dental care provider will refer the child for management by a dental specialist. When treatment needs are high, treatment is usually undertaken under a dental general anaesthesia (DGA) in the belief that it enables the provision of higher quality care than can be achieved in primary care settings [1, 2]. Parents have reported improved child oral health-related quality of life (COHRQoL) after treatment under a DGA [3]. Parents have also reported feelings of fear, worry, concern, and anxiety associated with their child undergoing a DGA [4, 5].

Recent research into the management of dental caries in primary teeth have suggested that dental care based on minimum intervention strategies can achieve good clinical outcomes among children and achieve similar impact on the child's oral health-related quality of life when compared with standard care interventions [6, 7]. Also, further recent findings suggest that minimal intervention approaches may allow successful management of early childhood caries and improve the COHRQoL of children initially assessed as requiring management of their oral condition under a DGA [8, 9].

Little is known about parent/carer (P/C) views on the acceptability of dental caries management of young children. Parents' views on the acceptability of three treatment approaches, including a minimally invasive approach to caries management was investigated in the United Kingdom (UK). The findings from that study suggest three principal factors were of importance; (1) Experience of the specific procedures; (2) Experience of anticipatory dental anxiety; (3) Effectiveness of the treatment, which were underpinned by a fourth factor of trust in the dental professional across the three approaches [10].

While there is some information on parent/caregiver perceptions regarding care under DGA [4, 5] there is no information, as far as the authors are aware, on P/C views comparing the management of their child's dental caries using alternative approaches with that of management under a DGA. A decision to undertake care under a DGA is usually taken once alternative non-DGA options have been explored, usually based on the extent of the required treatment and child/parent related factors [2, 11]. While acknowledging the use of other options such as pharmacological sedation and protective stabilisation in managing dental treatment of children [12], this study reports on P/C views within a randomised trial of the management of their child's dental caries under a DGA and an alternative minimally invasive approach using the Atraumatic Restorative Treatment and the Hall technique (ART/HT) after their child had been recommended a DGA. The findings with respect to clinical outcomes and impact on COHRQoL have been reported [8, 9]. The aim of this study was to elicit P/C views on approach to care with the DGA within a publicly funded care system and a minimally invasive alternative approach to the DGA.

## Materials and Methods

The core study was a randomised controlled trial (RCT; 32 ART/HT and 33 DGA) among children who were seen within a publicly funded dental specialty services provider in Western Australia (the Oral Health Centre of Western Australia, OHCWA). Ethical approval for the study was provided by the Princess Margaret Hospital for Children Human Research Ethics Committee and the trial registered with a clinical trials registry (HREC REF 2016143EP; ANZCTR: ACTRN12616001124426). The full details of the study have been reported [8]. Briefly, children who were seen within the specialist paediatric dentistry department of the OHCWA for the management of dental caries and who were advised to have a DGA for its management were invited to participate in the RCT by a project officer. Parents provided a signed informed consent to participate and completed a questionnaire. Children were then randomly allocated to either the DGA (control) or the ART/HT (test) arm by a different project officer to recruitment, using a computer-generated block randomisation procedure. Children allocated to the DGA arm are usually placed on a waiting list and are typically advised that if an emergency arise during the waiting period to seek care at the emergency department of the Children's Hospital, which will provide emergency care (generally, extractions only).

### Participant Recruitment

Sixty-five children participated in the RCT (mean age 4.7 years; mean dmft = 9.3). For the qualitative component of this study the recruiting project officer contacted participants in the randomised trial by telephone or personal approach at the final follow-up with a request to participate in a focus group interview. Attempts were made to obtain equal number of participants from each arm of the RCT. Parents who expressed an interest in participating in the focus group interviews were contacted again at the conclusion of the study to confirm their participation. Participants were advised that their agreement or refusal to participate has no bearing on their continued participation in the research project or their ongoing care at the OHCWA or other dental services. Each participant was provided with a store gift card to the value of $25 as compensation for their time.

All interviews were conducted by the recruiting project officer, who is a qualified oral health practitioner (HF) and have participated in conducting focus-group interviews with an experienced qualitative researcher. The interviews were in community facilities such as interview rooms within public libraries and community centres in close proximity to participant's place of residence, and some participants had their child participant or other younger child at the interview. Participants were informed that the interviews were to be audio-recorded and that written transcripts would be made of the recordings and that no identifiable individual identity would be reported. The recorded interviews were transcribed by an independent transcription agency. The interviewer also made copious notes after each interview sessions in relation to voice tone, non-verbal cues such as agitation, first responder to questions and comments on the general emotive atmosphere of the session.

### Interview Guide

The interviews were semi-structured and based around five broad open-ended questions, which were used as a guide to elicit P/C views on the care their child received. A sixth question was asked of each participant depending on their group allocation. All the interviewees were asked six guide questions, however, participants were free to canvass any other issues of importance to them in relation to their child's dental care. The open-ended questions were developed by an experienced qualitative researcher who undertook focus group interviews as part of an internal departmental project on the use of the Atraumatic Restorative Treatment (ART) for early childhood caries (ECC) in which parents' views on the use of for the management of ECC were explored [13]. The questions were also mailed to the participants prior to the interview along with their letter of invitation.

The questions asked were:

1. *What were some of the positive aspects of dental care your child experienced?*
2. *What were some of the negative aspects of the dental care your child received?*
3. *Can you give some examples of what you think could have been done/implemented better during your child's treatment?*
4. *Can you name some aspects of the setting/location/process that you think could have been improved?*
5. *Can you identify any changes to your oral health knowledge since the research began?*
6. *ART/HT participant; Can you tell me what your thoughts would be about your child's dental treatment if you had to pay for the treatment your child received?*
7. *DGA participant; Can you tell me what your thoughts would be about your child's dental treatment if an alternative to general anaesthetic was available, but you still had to pay for the treatment?*

### Analysis

Findings related to clinical outcomes and changes in child oral health-related quality of life have already been reported [8, 9]. The principal aim of this study was to develop an understanding of the P/C views on an alternative approach to the DGA with that of their views on managing their child's dental care needs and their valuing of how that care was delivered. It was not driven by theory development nor rooted in a particular framework of discourse analysis. To achieve the more utility aim of understanding the experiences of care users, we adopted a thematic analysis framework rooted in realist/experiential exploration of underlying themes to provide a rich and detailed account of the data. However, where emergent themes suggested a particular theoretical framework then these were explored [14]. The approach taken was to enable the findings to be applied in the development of policy and practice to improve the dental care of the young child and the thematic analytic framework to elucidate factors of importance to parents/carer was seen to be ideally suited for that purpose [15].

The transcribed notes were read numerous times independently by the two principal authors (PA, HF) and preliminary coding of the text undertaken and emergent themes identified inductively. Initial thematic development was undertaken with the readers making annotations on the transcripts and then developing a flow chart that identified commonalities and differences between the participants from the two groups. The coding was undertaken within the framework of the five principal questions. The analysts met several times to further develop the coding and identify emergent themes and where differences were encountered the matter was resolved through discussion and mutual agreement. The transcribed texts were imported into NVivo software and further refinement of coding and identification of emergent themes undertaken. The process was iterative and codes were added, eliminated or consolidated into other codes to assist in developing the emerging themes.

De-identified transcripts were further reviewed by the third author (SP) who was not associated with this research but have undertaken similar qualitative evaluation of another project. The three analysts subsequently met on a number of occasions (*via* teleconference) to review the findings and agree on the final themes and sub-themes. Relevant, illustrative comments from participants which conveyed and reflected their responses to the research question were then extracted from the transcripts.

## Results

**Figure 1:**
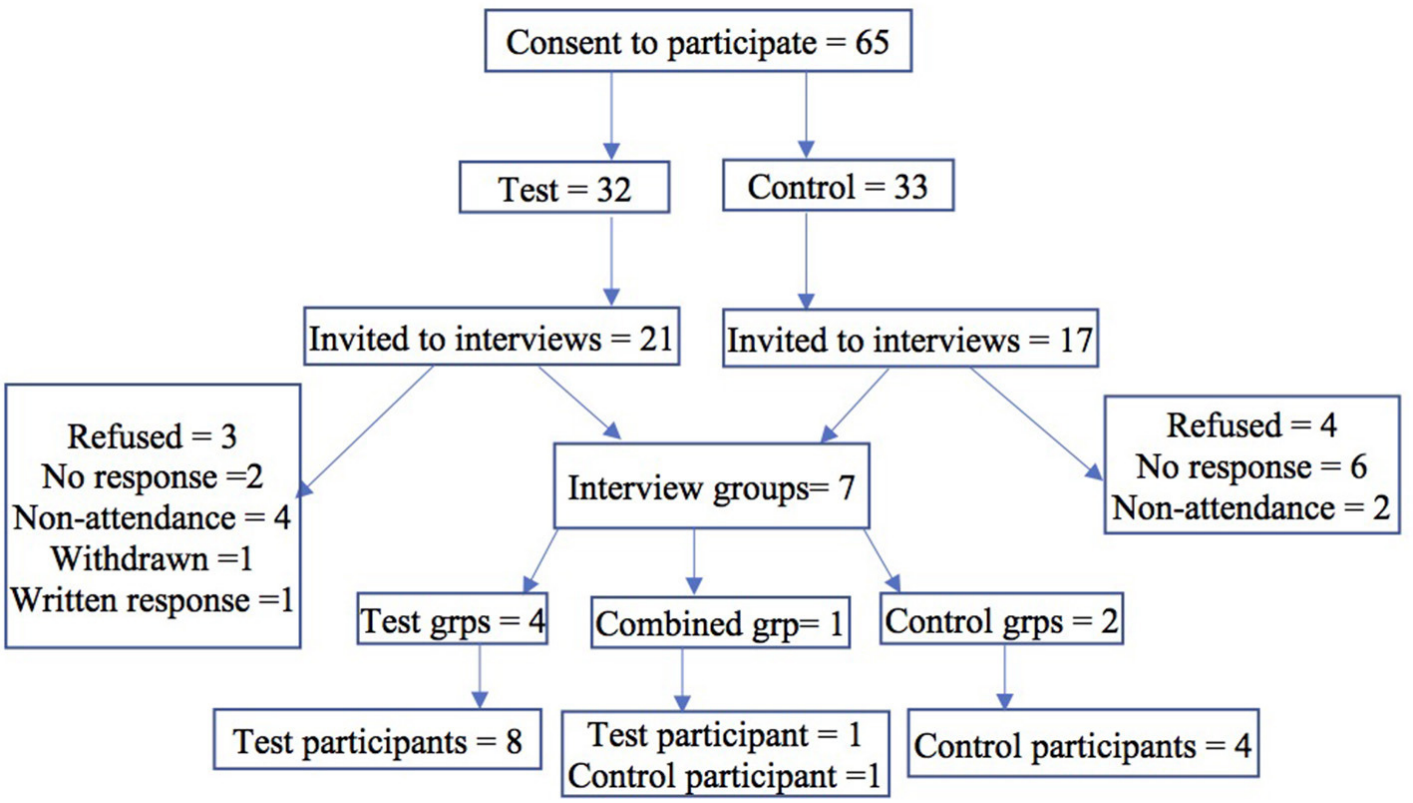
Participant flow chart.

Seven grouped interviews with 14 participants were conducted and one test participant provided a written response. Four groups with eight test participants; two groups with four control participants; and one combined group with one test and one control participant were interviewed between February and May 2019. Invitations were extended to parents who had indicated their willingness to participate *via* postal mail, e-mail, and phone text and confirmation text and reminder text the day prior to the interview date. Follow-up phone calls were also made to invite participation. The participant flow chart is shown in Figure 1. Of the 21 invitees from the test group ten participated (three of whom were fathers and two of the seven females were grandparents with primary carer responsibilities and others were mothers of the children). Of the 17 invitees from the control group five participated, all were mothers of the children. The participants were from diverse cultural backgrounds. The length of interviews ranged from 36 to 70 min.

Of the test group participants, one child was referred back for a DGA and one did not proceed with the ART/HT approach and the child received care under DGA. One child participant in the control group received care under conscious sedation. There was also a mix of previous experience with general anaesthesia (GA); seven in the test group had prior GA experience (four for dental and three for other reasons) while three participants in the control had prior GA experience (two for dental and one for other reasons).

In responding to the semi-structured questions all parents also reflected on their prior experiences of dental care and of GA experiences, either in WA or in their country of origin.

Parents willingly shared the extent of impacts felt by the child and the family (of the ECC and of the care; QoL, anxiety, treatment options). With respect to the care received, dominant themes that emerged were principally related to the process of care and were either viewed favourably or unfavourably. There were four themes related to treatment that emerged from both groups; (1) Evidence of child-/family-centred care; (2) Timeliness of care; (3) Affordable care; (4) Accessible care, with sub-themes within the dominant themes. Some themes were expressed more frequently in one group than the other. The themes and sub-themes related to ECC and treatments received are shown in Table 1.

**Table 1:** Elicited themes and sub-themes.

**Themes**	**Sub-themes**
Child and family impacts of ECC	Pain and functional limitations; psycho-social distress; fear and anxiety; treatment options
Child centred care/family centred care	Child anxiety; appropriate communication; dental extractions
Timeliness of care	Timely care
Affordable care	Access to care
Accessible care	Geographical location; care pathways; barriers to care

### Child and Family Impacts

P/Cs reported a broad range of impacts, which ranged from pain and functional limitations to psycho-social distress, experienced before and after treatment, not only by their child but also by themselves because of the ECC.

#### Pain and Functional Limitations

Mother ART/HT P10

…*like my daughter before is very bad pain have, and all the time he cry, miss the school, all the time not eating good, every time he's eating – all night I wake up just give him like …and all the time I can't sleep, myself, like I'm tired because when I see my daughter all the time cry … He's weight goes to 12 kilos*.

And after treatment;

*After treatment no I'm very happy like normal.… Yes it's now good, now 22 kilos*

Mother DGA P4

*Every morning he's crying because his teeth are sore, sore. He won't eat, he can't eat*.

And after treatment;

…*very big change. Now he got a little bit chubby now.… because it's already better so he sleep well now*.

#### Psycho-Social Stress

Mother ART/HT P2

*that was quite a lot to take on board when I was told all the work that he did need. I just felt really really bad as a parent*.

Mother DGA P5 (child treated under sedation)

*he put him in the chair and told me straight away, he had 16 cavities and he needed to have nearly every single tooth have a filling. …I cried, all the way home, … I don't know how I've done something so wrong…*

Grandmother ART/HT P7

*But his tooth decay itself …it did bother him*.

And after treatment;

*once they were repaired it made a huge difference in him, saying they're all nice, see my shiny teeth, he'd lift his lip up and show everybody*.

Mother DGA P4

*He got certificate for the grumpy kid*.

And after treatment

*But now his teacher is happy as a result. ……Yeah, and less stress too*.

P/C also reported of the limited discussion of the available options by their primary care provider and at the OHCWA.

Mother ART/HT P2

*it was just one option, there was no, we could try this or this or this, it was straight, it's the general anaesthetic because I can't get him to sit still to have an X-ray*.

Mother ART/HT P1

*We can't deal with her. Send her to the Oral Health Centre to get everything done*. … *because we've always been told that no-one can help her – she has to do it under general anaesthetic*.

And for some P/C, the option and the experience of having their child treated under a DGA was not viewed favourably and questioned the need for a DGA, and feelings of fear, anxiety and reluctance to have the child under GA were expressed:

Mother ART/HT P1

*For the general anaesthetic? Very anxious…*.

Father ART/HT P3

*we don't want to give him anaesthesia*

Mother ART/HT P9

*I really didn't want her under any anaesthetic, I was scared because she had grommets in her ears, when she went under I couldn't handle it, … It was too scary.… I had to hold her down as she went under it was just frightening, I was crying hysterical…*.

Mother ART/HT P11 (written response)

*(Child's name) had an experience to have teeth removed by putting her to sleep, she woke up confused and upset, and I personally don't think the IV sedation is no harm to children's health*.

While for some having all treatment done in the one visit was perceived to be a good option,

Mother DGA P2

*The reason I was probably thankful that we ended up going under GA is because the extent of work … So, getting that done in a chair would have been incredible multiple visits. It would have been tedious and long process. One of the reasons they also did it is like, well, everything's done at once, and you don't have to come back for years*.

And parents overrode their own fears of a DGA, in order for their child to have dental care:

Mother DGA P4

*Before, you know, as a mother, because I'm scared with the needle. … As a mother, I feel sorry, but it's for my son's sake, so he's feeling good after. It's okay*.

### Child-Centred Care

The concept of child-centred/family-centred care has been discussed extensively [16, 17] and for this study we have adopted the working definition by Ford, “*any approach to or philosophy of care which is characterized by positioning the child at the center of the care (whilst acknowledging importance of parents/carers and family)*” [18]. P/Cs of both groups expressed appreciation of the CCC when it was experienced.

Mother ART/HT P1

*Yeah, and I think the way (clinician's name) did it, she said, “Well, we'll work up to scary things like pulling the most painful teeth out”. So all that was done at the end when she was quite confident to go in. So, she started off with the smaller things and then worked up to fillings and things like that… Yeah, so dentist visits are now viewed as a positive experience.… Everyone involved was very approachable and non-judgemental…, ever since she was young, every time we went to see the dentist, they sort of made us feel like we were doing something wrong with her and that's why her teeth were like that……it didn't feel very rushed; … “It's a trust, yeah, to build that rapport.”*

Mother ART/HT P2

*For (child's name) it removed his nervousness as we went along because he was able to build up rapport with (clinician's name). It was really good. Initially he was quite apprehensive, but she was just very good in gauging how far to push him and I think he really like that he could tell her to stop and she would listen, so by the end of it he was like lets go see (clinician's name) and really happy which was wonderful and that wasn't the case in the beginning after his first visit it took some time to build up his confidence and from a parents point of view I feel, I know this is selfish, but I just feel so much better knowing that he has had the treatment that he needs without having to be completely knocked out, ……and knowing that he received the treatment and he is happy to go to the dentist feels good*.

Father ART/HT P3

*The good thing about that whatever time is convenient to me they managed to book it for me ……and it's a very warm welcome from everybody and … my son was totally engaged. (child) just laid on the chair, just hold it for me please, just give me a hand, you have got your sunnies, you know just keep engaging him, so then he feels like I am in safe hands. He had the procedure …… and my son was quite happy and he was really comfortable*.

Mother DGA P2

*The general anaesthetic part of it was awesome. … And yeah, they were really nice in the surgery and the recovery … and they really listened to what I requested. … and they only did what I felt was necessarily*.

Mother DGA P3

*But the biggest positive for us was…it was the first place I'd been to that didn't say, this is your fault. … They listened to me. So that was good, which made me more relaxed, which made him more relaxed. And they were all really good with making him relaxed…*

Mother DGA P5 (child treated under sedation)

*they did accept that we wanted to try in the chair, because I was a bit hesitant about the general anaesthetic… And I think they treated him with a lot of respect, and they were very gentle and kind with him, so I was very happy about that*.

However, parents were equally dissatisfied when child-/family centred care was absent, which was more commonly voiced by DGA parents.

Mother DGA P1

*They were really rude and mean. They basically pinned (child's name) down to stick the gas mask on her for her to be knocked out because she was hysterical. When she come out of the anaesthetic I wasn't in the room, they had woke her before I was even in there, so she was hysterical*. …

Father ART/HT (referred back for DGA) P5

*Being shunted really, without actually having anything done … It's, yeah, you get all set up, you get your expectations up, okay, he's going to get something done. And it's, oh, no, it's not going to happen now…. The staff at the hospital have been atrocious. They don't save the messages, they don't write anything down, appointments, we were there, you know, we're here, make an appointment, and they didn't even bother saving the appointment and we're sitting around until 5 o'clock in the afternoon, you know*.

Mother ART/HT P2 (at the specialist consultation)

*The assessment I …… but I felt like I was told it was my fault…*.

### Timeliness of Care

The extent of time spent waiting for a DGA was also a significant concern expressed by the participants. P/Cs found difficulty in reconciling what appears to be some urgency required to have the child treated and yet having to wait a substantial length of time.

Mother DGA P2

*But then they said, yep, we need to go under general and I waited six months patiently. I called up and no response, no response… And it was almost nine months before he had the surgery …*

Mother DGA P3

*When we got through to the Oral Health Centre it was, you're going to be waiting months and months*.

One parent who expressed a concern with the length of time to wait for treatment for her child reflected on her own experience within a public health system and was resigned to having to wait for care.

Mother DGA P4

…*I'm just a bit worried because his treatment is I'm waiting for long. I think it's one year. … because I understand, because it's public, you know…… so it's because my son is not emergency so it's okay for me to wait for that.……Yep because I used to – my stomach. I used to wait, even though I can't – because I got endoscopy before….…I'm waiting for a year as well. But sometimes I'm feeling I'm dying you know but I'm still waiting for one year*.

A similar concern was expressed by P/C in the ART/HT arm who were advised of the DGA option after their consultation at the OHCWA. Also, the provision of short-term temporary care when emergencies arise during the waiting period.

Mother ART/HT P2

…*and he is going on a waiting list for … and we can't do that for a year it didn't make sense to me if it was that important. I mean I understand the logistics and probably the demand on the system, but I thought my goodness what are we going to do for the next year then if it's that bad …… I thought it's imperative that he has this work done because he has all these issues with his teeth, and the poor little thing must be in a lot of pain*.

Father ART/HT P3

…*…we were just given an estimate and they said you are now in the queue I was surprised, I don't know when they are going to complete it, will it start aching more, …… he was having some sharp pain so he stopped eating and …he was not feeling well so immediately we rushed to the clinic …… we got an appointment for removal of his tooth. Then they said further to that you need to visit the Oral Health Centre and they will do the rest of them*.

### Affordable Care

Cost of dental care for their child was also a strong theme from P/C of both groups after the specialist consultation at the OHCWA.

Mother ART/HT P1

*I would have delayed completing the treatment … because money was quite tight, …*

Grandmother ART/HT P7

*I probably wouldn't have had it done, to the extent that it got done because I wouldn't have been able to financially afford it … he would not have got the treatment done*.

Mother DGA P1

*I think the bill nearly was $1,700 for her to have the operation. …… I still have $900 left, and then they won't see her until that bill's paid*.

Mother DGA P4

…*I'm worried because I think it is expensive. Because they told me it's probably $5,000*.

### Accessible Care

P/C of both groups were able to compare the ease/difficulty in physically getting to the location for their child to be treated. The principal issues identified by both groups were the distances involved in getting to the OHCWA, which is located in the city, and the challenges with trying to find parking.

Mother ART/HT P2

*I have to say the location was another big plus for me too, just not to have to go into Perth or into the city. In the morning just drop the other child to school, quickly take (child's name) and back again without having go to the city, it's quite stressful in itself getting into the city and finding parking on top of everything else*.

Mother ART/HT P9

*I didn't like the parking at the health centre (OHCWA), I was doing twenty laps in the car park trying to find a park. I didn't like the car park*.

Mother ART/HT P10

*it is hard to find parking, like sometime I missed the appointment about the parking*.

Grandmother ART/HT P7 reflecting on visiting the OHCWA and comparing it with visits at a local clinic for ART/HT care.

*to have to go there (OHCWA) for treatment on a weekly basis would've been extremely difficult … The appointment I had there was an early morning appointment… well that's like leaving two hours before. …So to be able to go out to (ART/HT treatment centre) was great. It was so convenient … take him to his 9 o'clock appointment. He was finished up there, he could go to kindy still, so it was good*.

Mother ART/HT P11 (written response)

…*…I didn't have to travel one and half hour each way to oral health centre……*

A sub-theme within accessible care was the frustration and confusion experienced by P/Cs in trying to negotiate the pathways to try and get care for their child. One parent indicated how it was the child health nurse who first identified the oral health problem and was on-referred by her medical practitioner to a dental clinic, who in turn referred her onto the OHCWA for care. While others reported of having to use different strategies to jump over the hurdles, or having to wait for a clinical emergency to arise to have care:

Mother DGA P1

*I physically went there with (child's name) myself, and demanded an appointment there and then, and I waited nearly two hours for an appointment and got seen, and then think it was eight weeks or maybe even longer before I got the appointment to (hospital name) for her operation*.

Mother DGA P3

*Through my mum's work, she actually managed to do the, I'm from such-and-such and I need you to push this through*.

Grandmother ART/HT P7

*I was not aware … at all to know where to or who to approach, and I did approach the primary school that I knew he was going to be attending, for them to tell me that sorry he's only four, we can't start treating him till they're at.Five, so that's why I went to a private … And then I just started looking around on the internet for what government services were available and that's where, I think I rang (government clinic). ……you had to be there at 8 o'clock in the morning. No appointment could be set at the time…… the system is you turn up there and it's first in first, we were there, we waited for a while and they just said look you will not be seen today … they then made me an appointment for another day.… and it was them who referred me to (OHCWA). They couldn't treat him because he was under five as well, apparently*.

Mother DGA P3

*We literally would have had to wait for something to go wrong… child abscesses their teeth or whatever, and then they get seen straight away*.

A related concern was, what was felt to be, radical recommendations for dental extractions at the specialist consultation, and at treatment under DGA.

ART/HT 4 (parent opted for DGA)

…*and he took four out but that's the disappointing bit, … I was advised by the surgeons that there's an extra tooth so need to remove that…but when he's come out from the surgery and four teeth was missing … it was a shock to me…*

Mother DGA P1

*They removed more teeth than what they said they were going to. …… So she only ended up with her fangs up the top and all her other teeth removed*.

And parents were relieved when teeth could be treated more conservatively and the extractions were able to be avoided;

Mother ART/HT P2

…*… I was told initially, they would just pull out as a precaution are now still in place and looking very healthy.… and he has none removed at the moment. She did say we don't know about the molars whether they will have to be eventually but I think three other teeth were saved*.

For the P/C who had their child managed under ART/HT, they were pleasantly surprised, not only that treatment was able to be done but the extent of the treatment, which sometimes included extractions:

Mother ART/HT P8

*The school dentist wouldn't touch her because she is terrified of the dentist … a private dentist and they wouldn't touch her, so thank God for (clinician's name) … and (child's name) settled within the first two visits. I could not believe she had done all that work and (child's name) just laid there and let her do it. It was amazing*.

Mother ART/HT P1

*I was surprised because from not being able to even open her mouth to letting them put her tooth to sleep and pull it out…*

Grandmother ART/HT P7

*Well the dentist I'd seen privately would not consider it in any other way (DGA). Then through (OHCWA) I don't think that really was even discussed, you know. And I think it was great that he was able to have the treatment without that, anaesthetic and no needles, no anything*.

And parents who have had previous experience with a GA, either with their child in the study or another child, and were managed through the ART/HT were very satisfied with the care received and even expressed a willingness to pay for the care:

Mother ART/HT P8

*It was going to cost money either way you go, I would rather pay (clinician's name) than pay somebody to knock her out and do it…*.

Father ART/HT P3

*Absolutely. I was mentally prepared. Either it's on the chair or under GA, so I have to pay. …It's always better to get it done as soon as possible*.

Mother ART/HT P2

…* either way we are going to have to pay. … so to be able to pay for treatment that he has in the chair and he still comfortable and confident to go to the dentist I think it's all you need.… I just feel so much better knowing that he has had the treatment that he needs without having to be completely knocked out…*.

While the P/C in the DGA arm felt that a DGA was the only viable option for their child:

Mother DGA P1

*No other option… because we have trouble just going to dentists now… She won't hold her mouth open for long … so there is no other option for (child's name) but to be put under*.

Mother DGA P3

*We went through them because my son has such a bad gag reflex they could not even get the x-ray*.

## Discussion

Dominant themes to emerge from the interviews were the impacts of ECC on the child and the family, the receipt of child-centred care, coupled with receipt of timely care, and the provision of appropriate information. Although, the aim of the study was to ascertain the perspectives of P/C on the way their child was provided with dental treatment, guided by semi-structured questioning, our participants expressed wide-ranging views on ECC and its treatment. The participants in the study were parents and carers of children who took part in a two-arm RCT to test a minimally invasive, atraumatic approach to manage ECC. Thus, we were able to obtain the views of parents whose child was managed under a DGA as well as the views of those whose child was managed using the atraumatic approach. The responses provided indicated that the thematic analysis was able to elicit the views comprehensively.

Participants were initially seen by a specialist at a tertiary specialty centre that provided dental treatment to people who were eligible for a means tested government subsidised care. Hence, the responses are specific to specialist care within a public facility at a point in time and are not necessarily applicable to other settings. All participants had been referred for specialist consultation by their primary care provider and had been recommended for care under a DGA after the specialist consultation. Parents from both groups were also offered limited options other than a DGA for the management of their child's dental care by their primary care provider.

The P/C reported significant impacts on the child and the family as a result of ECC. Information obtained *via* questionnaires have indicated the significant impact of dental caries on the child and the family [9, 19, 20]. The personal stories told by our participants further underlie the significant impacts felt by P/C and children as a result of dental caries, such as a child's capacity to function holistically in being pain free and being able to eat, sleep, and interact socially and the flow-on impacts on the family. Similar reports of child and family impacts of a child's inability to function and socially interact meaningfully and parental distress have been found in studies elsewhere [5, 21]. In the Brazilian study parents reported impacts relating to pain on eating, mispronunciation of words, and not wanting to smile for photos and parental guilt. Similarly, the parents in the study by Lee et al. reported feelings of being a bad parent. PC of both groups in our study reported equally of positive impacts on the child and the family as a result of care received, but feelings of frustration was also expressed when care could not be obtained in a timely manner.

The dominant theme to emerge with respect to receipt of dental care was the central role of child- and family-centred care in service delivery. More P/C in the ART/HT arm reported experiences of CCC. The conceptualisation of CCC as one in which the child is seen as social being fully able to participate meaningfully and engage in the care process is seen as central to delivering appropriate care to children [16, 22]. The settings for using the ART/HT approach where there are multiple treatment visits provided greater opportunities for the necessary engagement with the child and parent/carer to establish rapport and trust between the clinical team and the child and enabled the delivery of CCC [23, 24]. The CCC emphasises patient care rather than disease-centred care and is a core component in evaluating quality of care, and trust has a pivotal role in establishing the care alliance [25]. The establishment of trust as a core component of acceptance of treatment has been identified in the UK study which tested three alternative approaches to manage dental caries in primary molars of children [10]. The authors of the UK study suggested that the establishment of trust enabled a treatment alliance between the clinician and the child and parent which in turn assisted in acceptance of the treatment.

Dissatisfaction with care when CCC was perceived to be absent has been reported [26]. The delivery of CCC can commence from the initial contact with P/C and child, even before they present to the clinic, and care delivery was enhanced when these opportunities were taken. Irrespective of the child receiving care from either the ART/HT approach or a DGA, both groups of P/C in general were satisfied with the care provided, but concern was expressed in being able to obtain timely and affordable care for their child. P/C were generally accepting of having to wait for care, but for some the need to negotiate multiple hurdles was frustrating and led to significant anger and disenchantment and may have been a factor in the relatively poorer response by the DGA group to focus group invitations.

Management under DGA tends to be more aggressive in order to mitigate against post-DGA complications [27] and teeth for which conservative management may have been attempted in a primary care setting are more likely to be extracted under a DGA. The P/C in general had difficulty in accepting dental extractions and expressed a wish for greater consultation and information when extractions were planned and undertaken. The impact on the child of dental extractions treatment may be negative [4], but this was not universally reported by our participants.

It was also apparent that P/C were not fully advised of the alternative options available to DGA, either because the primary care provider was not aware of the options or was not comfortable with providing the alternative care. P/C of both groups expressed fear and concerns with a DGA, which have been echoed in other similar qualitative studies [4, 5], but overcame those fears in order to obtain care for their child. For some parents, treatment under GA was viewed as the only option because of the difficulties their child posed in accepting treatment, while for others, alternative treatment options such as sedation or guided management were seen as options. The findings suggest that both options of ECC management were acceptable but that other issues of concern were accessibility, timeliness, and affordability associated with public dental care for specialty paediatric dental services.

Evaluation of P/C views on different approaches to the management of ECC was important to assess its consistency with clinical and COHRQoL findings of the core RCT [8, 9]. The core study found that children recommended for a DGA could be successfully provided with dental treatment in a primary care setting using the ART/HT approach and the treatment approach also improved the COHRQoL. The major factor of importance in care delivery reported by P/Cs was the evidence of CCC, especially when the child was involved in the care process. However, P/Cs also identified significant barriers within the public dental service framework in obtaining the needed care for a child with ECC.

The limitations within this qualitative evaluation include the fact that the P/Cs who took part in the interviews were not a random sample but were those who were willing and wished to share their information. All were also eligible for government subsidised care in Western Australia, thus can be considered as socio-economically disadvantaged. There was a lower response rate from the DGA arm to the focus group interviews, which may have been as a result of poor experience with the public dental care system, and not necessarily due to the DGA *per se*. However, the DGA experience is encapsulated within the system which the P/C have to negotiate in order to access care for their children, thus, can be considered a real-world representation within the WA public dental services, but may not be representative of the situation in other states and territories in Australia nor in non-public settings. At the time of the study being undertaken there were also structural and system changes occurring which may have impacted on access to DGA care, such as availability of theatre time for DGAs and relocation of the treatment facility to the new Perth Children's Hospital.

## Conclusion

P/Cs of children with ECC who participated in a trial to test an alternative approach to that of a DGA for the management of their child's caries generally perceived the alternative minimally invasive approach positively. The experience of timely, and CCC was of importance to parents and carers and positive impacts were reported where care had been received in such a manner irrespective of whether it was under GA or with the ART/HT approaches. The findings suggest that minimally invasive approaches underpinned by child-centred care tenets are acceptable alternative options to the DGA and should be considered for the management of ECC. P/Cs also experienced significant challenges in negotiating the pathways to obtain accessible, affordable, and timely care for their child within public dental services.

## Data Availability Statement

The datasets presented in this article are not readily available because data are not available for distribution. Requests to access the datasets should be directed to parrow@ozemail.com.au.

## Ethics Statement

The study involving human participants was reviewed and approved by Princess Margaret Hospital for Children Human Research Ethics Committee. Written informed consent to participate in this study was provided by the participants' legal guardian/next of kin.

## Author Contributions

All authors listed have made a substantial, direct and intellectual contribution to the work and approved it for publication.

## Conflict of Interest

The authors declare that the research was conducted in the absence of any commercial or financial relationships that could be construed as a potential conflict of interest.
